# Update: Ebola Virus Disease Epidemic — West Africa, January 2015

**Published:** 2015-02-06

CDC is assisting ministries of health and working with other organizations to end the ongoing epidemic of Ebola virus disease (Ebola) in West Africa (1). The updated data in this report were compiled from situation reports from the Guinea Interministerial Committee for Response Against the Ebola Virus, the Liberia Ministry of Health and Social Welfare, the Sierra Leone Ministry of Health and Sanitation, and the World Health Organization.

According to the latest World Health Organization update on January 28, 2015 (3), a total of 22,092 confirmed, probable, and suspected cases of Ebola and 8,810 deaths had been reported as of January 25 from the three West African countries (Guinea, Liberia, and Sierra Leone) where transmission has been widespread and intense. Total case counts include all suspected, probable, and confirmed cases, which are defined similarly by each country (2). Because of improvements in laboratory diagnostics and surveillance, in recent weeks totals may overestimate the actual number of cases in some areas. The highest reported confirmed case counts were from Sierra Leone (7,968) and Liberia (3,138), followed by Guinea (2,569). During the week ending January 24, an average of 11 confirmed cases were reported from Sierra Leone, less than one from Liberia, and three from Guinea each day. The areas with the highest number of confirmed cases reported during January 5–25 were the Western Area and Port Loko, Sierra Leone (Figure).

Eight cases and six deaths were previously reported from Mali (4,5). No new confirmed cases have been reported from Mali since December 5, 2014. On January 18, 2015, the World Health Organization declared Mali free of Ebola (6).

The latest updates on the ongoing Ebola epidemic in West Africa, including case counts, are available at http://www.cdc.gov/vhf/ebola/outbreaks/2014-west-africa/index.html. The most up-to-date infection control and clinical guidelines for the Ebola epidemic in West Africa are available at http://www.cdc.gov/vhf/ebola/hcp/index.html.

## Figures and Tables

**FIGURE:**
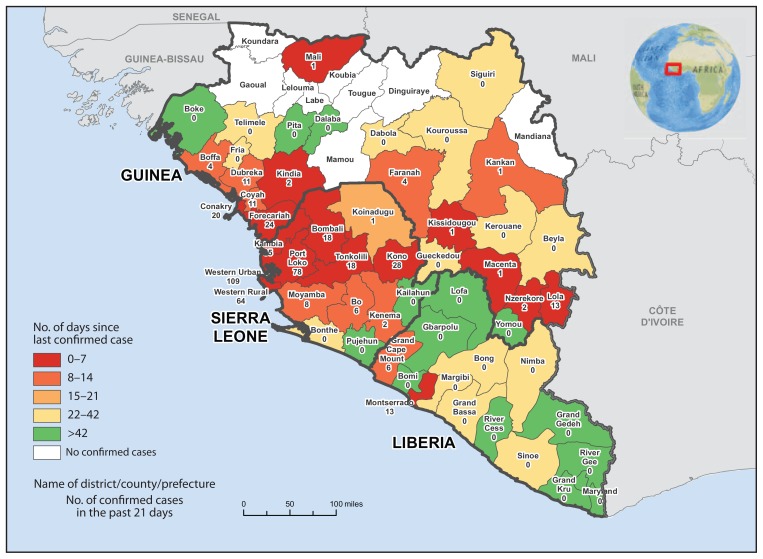
Number of days since last confirmed case of Ebola virus disease and number of confirmed cases in the past 21 days — Guinea, Liberia, and Sierra Leone, January 5–25, 2015* **Sources:** Guinea Ministry of Health; Liberia Ministry of Health and Social Welfare; Sierra Leone Ministry of Health and Sanitation; World Health Organization. *Data as of January 25, 2015.
